# Comparison of Callisphere Drug-Eluting Beads Transarterial Chemoembolization and Conventional Transarterial Chemoembolization for the treatment of Hepatocellular Carcinoma

**DOI:** 10.12669/pjms.40.3.8572

**Published:** 2024

**Authors:** Yongjun Ren, Caixia Zhao, Yongfu Xiong, Zhongbai Liu, Guo Wu

**Affiliations:** 1 Yongjun Ren, Department of Hepatobiliary Surgery, Affiliated Hospital of North Sichuan Medical College, Nanchong, Sichuan Province 637000, P.R. China; 2 Caixia Zhao, Department of Oncology, Nanchong Central Hospital, Nanchong, Sichuan Province 637000, P.R. China; 3 Yongfu Xiong, Department of Hepatobiliary Surgery, Affiliated Hospital of North Sichuan Medical College, Nanchong, Sichuan Province 637000, P.R. China; 4 Zhongbai Liu, Department of Hepatobiliary Surgery, Affiliated Hospital of North Sichuan Medical College, Nanchong, Sichuan Province 637000, P.R. China; 5 Guo Wu, Department of Hepatobiliary Surgery, Affiliated Hospital of North Sichuan Medical College, Nanchong, Sichuan Province 637000, P.R. China

**Keywords:** CalliSpheres, Transarterial chemoembolization, Drug-eluting beads transarterial chemoembolization, Conventional transarterial chemoembolization, Hepatocellular carcinoma

## Abstract

**Objective:**

To compare the efficacy of CalliSphere drug-eluting beads (DEBs) and conventional (c) transarterial chemoembolization (TACE) in the treatment of hepatocellular carcinoma (HCC).

**Methods:**

We retrospectively reviewed the clinical data of 125 patients with HCC who had received treatment in Affiliated Hospital of North Sichuan Medical College from January 2018 to February 2019. Sixty-one patients underwent DEB-TACE (observation group) and 64 patients underwent cTACE (control group). The clinical efficacies, overall survivals, and incidence of postoperative adverse reactions between the two groups were compared.

**Results:**

The objective response rate in the observation group (85.25%) was higher than that in the control group (70.31%; *P*<0.05). The disease control in the observation group (96.72%) was higher than that in the control group (85.94%; *P*<0.05). The median survival time of the observation group (24.85 months) was significantly higher than that in the control group (18.18 months; *P*<0.05). The incidence of adverse reactions in the observation group (4.92%) was lower than that in the control group (17.19%, *P*<0.05).

**Conclusions:**

In the treatment of HCC, Callisphere DEB-TACE has better efficacy and longer patient survival with fewer adverse reactions compared to cTACE.

## INTRODUCTION

Primary hepatocellular carcinoma (HCC) is a prevalent malignant tumor^1^ and a leading cause of cancer-related deaths worldwide, ranking sixth and third, respectively.^1^,^2^ Surgical tumoral resection is the most effective treatment for HCC, but the lack of specific symptoms in the early stages of HCC often leads to diagnoses at middle to late stages with portal vein tumor thrombi or distant metastasis, resulting in a missed opportunity for surgical intervention.^3^ Many treatment methods are available for such patients in clinical practice (including systemic chemotherapy and radiotherapy), but their overall effects are not ideal.^3^,^4^

Transarterial chemoembolization (TACE) has become the preferred treatment option for unresectable HCC.^5^ It shrinks or eradicates tumors by targeting them and blocking their blood flow and locally injecting chemotherapy drugs to the tumor, which promotes ischemic necrosis of the tumor tissue.^6^ The ideal TACE achieves precise embolization of the supply artery delivering the maximum concentration of chemotherapy drugs into the tumor lesion.^5^,^6^ Thus, choosing an appropriate embolization drug is important. Iodized oil, with its ability to embolize both hepatic arteries and portal veins, has been a commonly used embolic agent with good tumor tropism and drug carrying plasticity.^7^ However, its high fluidity and weak drug binding lead to poor deposition and may result in drug entry into the systemic circulation, and decreased therapeutic efficacy, potentially increasing the risk of adverse reactions.^7^,^8^ The development of drug-loaded microspheres has offset the shortcomings of iodized oil, allowing for targeted selection of blood vessels, better compatibility with blood vessels, precise and thorough embolization, and slow release of chemotherapy drugs, while maintaining high concentrations of drugs in the tumor area, reducing drug concentrations in the systemic circulation, and improving outcomes with fewer adverse reactions.^9^

At present, the choice between the Callisphere drug-eluting beads transarterial chemoembolization (DEB-TACE) and conventional TACE (c-TACE) in clinical practice is primarily determined by the attending practitioner, and there is no standard protocol for their application. 7–9 In recent years, studies have been conducted to compare the treatment response, survival and safety between Callisphere DEB-TACE and c-TACE.^10^-^12^ However, the findings of the studies were not consistent. Therefore, we compared the effectiveness of DEB-TACE and c-TACE in patients with HCC to provide more evidence for previous studies.

## METHODS

We retrospectively reviewed the medical records of 125 patients with HCC treated at Affiliated Hospital of North Sichuan Medical College from January 2018 to February 2019. Sixty-one patients underwent DEB-TACE (observation group) and 64 patients underwent cTACE (control group).

### Inclusion criteria:

- Patients ≥ 18 years old
- Patients with confirmed diagnosis of primary HCC^13^
- Patients received DEB-TACE using CalliSpheres microspheres (CSM) or conventional TACE (cTACE).
- Patients with Child Pugh liver function grade A or B, without symptoms of decompensated liver cirrhosis
- Patients without history of target lesion surgical resection, radiofrequency ablation, or chemotherapy
- Patients with complete medical records and follow-up information

### Exclusion criteria:

- Patients presenting diffuse growth or metastasis of intrahepatic tumors
- Patients with concomitant active hepatitis
- Patients with severe organ dysfunction of heart, liver, or lungs

### Ethical Approval

All procedures conducted during the research involving human participants complied with the ethical standards of institutions and/or national research committees, as well as the Helsinki Declaration (revised in 2013). The Medical Ethics Committee of Affiliated Hospital of North Sichuan Medical College approved this study (No. 2022ER387-1, Date: 2022-10-12).

The patients in the control group received cTACE while the patients in the observation group received Callisphere DEB-TACE. The treatment protocol referred to previous literature and briefly described as below.^10^,^12^

### cTACE (control group)

The surgeon first mixed 150-200 mL of 5% glucose injection (Sichuan Kelun Pharmaceutical, H51020634) with 100 mL of oxaliplatin (Jiangsu Hengrui Pharmaceutical, H20050962) and then slowly injected the solution into the target vessel over a 20-minute period. Subsequently, the surgeon injected 20 mg of epirubicin (Zhejiang Haizheng Pharmaceutical Co., Ltd., National Approval No. H19990280) dissolved into an appropriate amount of iodized oil (Yantai Luyin Pharmaceutical, National Approval No. H37022398) into the tumor’s blood supply vessel under fluoroscopy. The injection was stopped once the flow of the oil became stagnant or displayed reflux.

### Callisphere DEB-TACE (observation group)

Drug sustained-release microspheres, chemotherapy drugs, and contrast agents were injected at a controlled rate of one mL/minute. Embolization was performed sequentially on each tumor blood supply vessel on the basis of each patient’s specific case. Once a stagnant state was reached, the tumor was kept stationary for five minutes before re-imaging. If the tumor showed staining, additional embolization was performed until the endpoint of embolization was achieved (no emptying of the contrast agent after 3-4 cardiac cycles). The 100–300-µm drug-loaded microspheres used in the treatment were purchased from Suzhou Hengrui Jialisheng Biomedical Technology. The operator drew the drug-loaded microspheres and buffer solution into a 20-mL syringe, placed it vertically, and allowed it to stand for one minute before discharging the supernatant. To prepare the drug sustained-release microspheres, the operator first extracted 3mL of 5% glucose and diluted 60mg of epirubicin into a syringe, with a concentration controlled at 20mg/mL. A three way valve was then connected, and the drug-loaded microspheres were mixed with the glucose-epirubicin solution and allowed to absorb the drug for 30 minutes. Following this, 5–10mL of non-ionic contrast agent were injected until the microspheres were evenly suspended in the syringe. Finally, each bottle of microspheres was loaded with 60 mg of epirubicin, and pulse injection embolization was selected.

### Clinical efficacy evaluation

At each follow-up, we assessed the treatment’s efficacy using the modified response evaluation criteria in solid tumors (mRECIST)^14^: The disappearance of arterial enhancement in the target tumor resulted in complete response (CR); a reduction of 30% or more in the total diameter of the arterial enhancement area within the target tumor corresponded to a partial response (PR); a decrease in the total diameter of the arterial enhancement area within the target tumor of less than 30%, or increases of less than 20% indicated stable disease (SD); and, an increase by 20% of the total diameter of the arterial enhancement area within the target tumor, or the appearance of new lesions were considered progressive disease (PD). We used the following formulas to calculate the objective response and control rates: objective response rate = (CR cases + PR cases)/total cases × 100%; and disease control rate = (CR cases + PR cases + SD cases)/total cases × 100%. We surveyed the occurrence of adverse reactions in patients, including those like pain, nausea and vomiting, abdominal pain, fever, liver abscess, liver rupture, gastrointestinal bleeding, and liver dysfunction. The final follow-up was set at February 2023 or patient death.

### Statistical Analysis

We conducted all data analyses using SPSS 25.0 software. Continuous data were expressed as means ± standard deviations and compared using student’s t tests. Counting data were expressed by number of cases and compared using chi-square test. We applied the Kaplan Meier method to plot the survival curves, and the Log rank test to compare the overall survival periods. We considered all *P*<0.05 as reflecting a statistically significant difference.

## RESULTS

**Fig.1:**
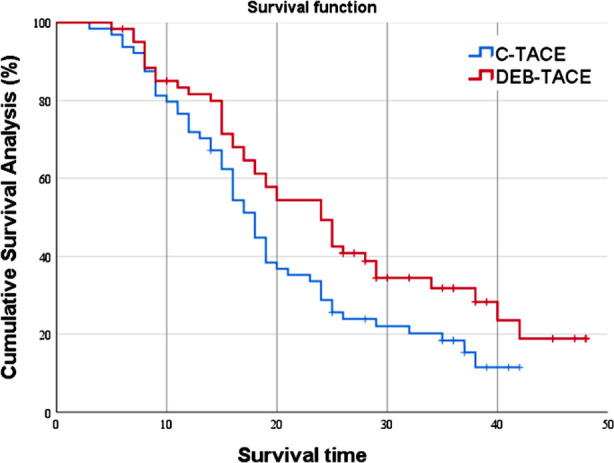
Comparison of survivals between the two groups.

In this study, we reviewed the records of 125 patients (85 men and 40 women) with ages ranging between 39 and 73 years (average, 57.91 ± 7.60 years). Their tumor diameters ranged between 5.4 and 18.9 cm (average, 12.15 ± 3.65 cm); and their Child Pugh liver function grades were A in 41 cases and B in 84. The average hospitalization length was 5.02 ± 1.30 days; 64 patients received cTACE, and 61 received DBE-TACE treatments. We found similar values for background information variables such as gender, age, and tumor status in the two groups (P>0.05; Table-I). The objective remission rate in the observation group at 85.25% was higher than that in the control group at 70.31% (P<0.05), and the disease control rate in the observation group (96.72%) was higher than that in the control group (85.94%, P<0.05) (Table-II). The median survival time of the observation group was 24.85 months, significantly higher than the 18.18 months of the control group (χ^2^=4.546; P=0.033) (Fig-1). The incidences of adverse reactions were 4.92% in the observation group and 17.19% in the control group (P<0.05, Table-III).

**Table-I:** Comparison of general data between the two groups

Group	n	Gender (Male/Female)	Age (years)	Tumor diameter (cm)	Child-Pugh liver function grading		Hospitalization duration (days)

					A	B	
Control group	64	41/23	58.69±6.95	11.76±3.77	23	41	5.16±1.26
Observation group	61	44/17	57.10±8.21	12.57±3.50	18	43	4.89±1.33
*χ^2^*/*t*		0.934	1.170	-1.245	0.586		1.168
*P*		0.334	0.244	0.215	0.444		0.245

**Table-II:** Comparison of clinical efficacies between the two groups.

Group	n	Curative effect				Objective response rate	Disease control rate

		CR	PR	SD	PD		
Control group	64	10 (15.63)	35 (54.69)	10 (15.63)	9 (14.06)	45 (70.31)	55 (85.94)
Observation group	61	21 (34.43)	31 (50.82)	7 (11.48)	2 (3.28)	52 (85.25)	59 (96.72)
*χ^2^*						4.526	4.635
*P*						0.033	0.031

**Table-III:** Comparison of adverse reactions between the two groups [n (%)].

Group	n	Adverse reactions								

		Pain	Nausea and vomiting	Abdominal pain	Fever	Hepatic abscess	Hepatic rupture	Gastrointestinal hemorrhage	Liver dysfunction	Total
Control group	64	1 (1.56)	2 (3.13)	1 (1.56)	1 (3.13)	1 (1.56)	2 (3.13)	1 (1.56)	2 (3.13)	11 (17.19)
Observation group	61	0 (0)	1 (1.64)	0 (0)	1 (1.64)	0 (0)	1 (1.64)	0 (0)	1 (1.56)	3 (4.92)
*χ^2^*	-	-	-	-	-	-	-	-	-	4.727
*P*	-	-	-	-	-	-	-	-	-	0.030

## DISCUSSION

The results of this study showed that Callisphere DEB-TACE is more effective than cTACE in treating patients with HCC, with longer survival time and fewer adverse reactions. Wang D et al^15^ showed that the objective response rate of patients with HCC treated with drug-loaded microspheres was significantly higher and the incidence of adverse reactions lower than those of patients treated with iodized oil. Zhang X et al^16^ conducted a prospective cohort study of 367 patients and found that the DEB-TACE treatment has the same efficacy as cTACE and is tolerated just as well by patients. Those results are consistent with ours. In a retrospective study, Ye T et al^17^ found that patients undergoing DEB-TACE exhibited a higher subjective tolerance to the treatment for advanced HCC with high tumor burden than those undergoing cTACE; additionally, the DEB-TACE treatment led to a reduction in the number of interventional treatments needed. Zou JH et al^18^ screened studies published from January 1990 to March 2015 and reviewed parallel group designs comparing cTACE and DEB-ACE for treating HCC, their results showed that DEB-TACE led to a higher complete response rate and overall survival rate than cTACE, in addition to presenting a safer profile and resulting in fewer adverse events. Our research is consistent with the above findings. However, both Zhao et al^10^ and Xiang et al^12^ found that the safety profile of Callisphere DEB-TACE were equivalent compared to cTACE, which is inconsistent with our findings. We believe that different sample size and patient characteristic may contribute to the discrepancy.

Our results also showed that the patients in the DEB-TACE group had a higher survival than those in the cTACE group. The drug-loaded microspheres used in this study are synthetic; their skeleton is composed of polyvinyl alcohol cross-linked with reactive blue by means of covalent bonds. The material is non-biodegradable and can be loaded with a variety of chemotherapy drugs to permanently block target vessels for the treatment of liver cancer.^9^,^19^ The microspheres are rich in anions, which combine with positively charged chemotherapy drugs to form ionic bonds.^19^ After entering the human body, the drug molecules compete with other ions in the body’s fluids, and the chemotherapy drugs get released, effectively cutting off the tumor blood supply and accurately killing the tumor cells.^19^,^20^ Drug-loaded microspheres have the advantages over iodinated oil as an embolic agent: They have a uniform particle size and smooth surface, which allows them to accurately target blood vessels for embolization. In addition, they possess good variable elasticity and can perfectly fit blood vessels for complete embolization. The results by Si Y et al^21^ suggested that the ideal TACE protocol maintains a maximally sustained concentration of chemotherapy drugs in the tumor, achieving an ideal combination of supply vessel occlusion and local exposure. Iodized oil and drug emulsification are temporary preparations, and their dosage is determined on an individual’s patient basis during surgery. Non standardized procedures do not always achieve complete embolization of blood vessels, and they may even result in weak drug binding, accelerated drug release, and increased incidence of adverse reactions.^6^,^21^ The extremely small diameter of drug-loaded microspheres can completely embolize the tumor’s blood supply vessels while slowly and continuously releasing chemotherapy drugs within the tumor and maintaining long-term and high-level drug concentrations, to further improve the therapeutic efficacy, and reduce the toxic side effects of chemotherapy drugs and any possible adverse reactions.^9^,^19^,^22^ However, the same small diameter of the drug-loaded microspheres may cause collateral channel blocks while embolizing target blood vessels, leading to ischemic necrosis of the adjacent liver, thereby reducing liver function.^23^,^24^ We did not find any worsening of the liver function in the patients receiving drug-loaded microsphere treatments, and further research is needed in this regard.

On the basis of our results, we propose that a comprehensive evaluation of the patient’s overall and tumoral conditions should be carried out, and that embolization drugs need to be carefully selected before conducting a hepatic artery chemoembolization for the treatment of HCC. If necessary, drug-loaded microspheres and iodinated oil can be combined to further enhance the clinical benefits for patients.

### Limitations

The sample size of this single-center retrospective analysis was small. Moreover, only a few variable indicators were assessed in this study. Therefore, the conclusions of this study may have a certain degree of subjectivity.

## CONCLUSION

In the treatment of HCC, Callisphere DEB-TACE has better efficacy and longer patient survival with fewer adverse reactions compared to cTACE.

### Authors’ contributions:

**YR and CZ** conceived and designed the study.

**YX and ZL** and GW collected the data and performed the analysis.

**YR and CZ** were involved in the writing of the manuscript and is responsible for the integrity of the study.

All authors have read and approved the final manuscript.
